# MiR-449a Affects Epithelial Proliferation during the Pseudoglandular and Canalicular Phases of Avian and Mammal Lung Development

**DOI:** 10.1371/journal.pone.0149425

**Published:** 2016-02-18

**Authors:** Ethan L. Sanford, Kwong W. Choy, Patricia K. Donahoe, Adam A. Tracy, Regis Hila, Maria Loscertales, Mauro Longoni

**Affiliations:** 1 Pediatric Surgical Research Laboratories, Massachusetts General Hospital, Boston, MA, United States of America; 2 Health Sciences and Technology Medical Program, Harvard Medical School, Boston, MA, United States of America; 3 Department of Medicine, Boston Children's Hospital, Boston, MA, United States of America; 4 Department of Obstetrics & Gynaecology, The Chinese University of Hong Kong, Hong Kong, China; 5 Department of Surgery, Harvard Medical School, Boston, MA, United States of America; 6 Broad Institute of MIT and Harvard, Cambridge, MA, United States of America; University of Southern California, UNITED STATES

## Abstract

Congenital diaphragmatic hernia is associated with pulmonary hypoplasia and respiratory distress, which result in high mortality and morbidity. Although several transgenic mouse models of lung hypoplasia exist, the role of miRNAs in this phenotype is incompletely characterized. In this study, we assessed microRNA expression levels during the pseudoglandular to canalicular phase transition of normal human fetal lung development. At this critical time, when the distal respiratory portion of the airways begins to form, microarray analysis showed that the most significantly differentially expressed miRNA was miR-449a. Prediction algorithms determined that N-myc is a target of miR-449a and identified the likely miR-449a:N-myc binding sites, confirmed by luciferase assays and targeted mutagenesis. Functional *ex vivo* knock-down in organ cultures of murine embryonic lungs, as well as *in ovo* overexpression in avian embryonic lungs, suggested a role for miR-449a in distal epithelial proliferation. Finally, miR-449a expression was found to be abnormal in rare pulmonary specimens of human fetuses with Congenital Diaphragmatic Hernia in the pseudoglandular or canalicular phase. This study confirms the conserved role of miR-449a for proper pulmonary organogenesis, supporting the delicate balance between expansion of progenitor cells and their terminal differentiation, and proposes the potential involvement of this miRNA in human pulmonary hypoplasia.

## Introduction

Abnormal lung development, often presenting at birth as functional respiratory distress, is a prominent feature associated with Congenital Diaphragmatic Hernia (CDH) [1,2]. Anatomically and histologically, pulmonary embryology has been traditionally divided into five successive stages. In the first two, the embryonic and the pseudoglandular stages, the conducting airways are formed. The later canalicular, saccular, and alveolar stages are characterized by epithelial differentiation, increased vascularization, compaction of the mesenchyme, and maturation of the air-blood interface in the alveoli [3–5]. At birth, the respiratory tree contains several specialized epithelial cell types, organized along its proximo-distal axis; these include the ciliated, secretory, and neuroendocrine cells in the proximal bronchi, and type I/II pneumocytes in the distal alveoli [3]. Key morphogenetic events are common between all vertebrates, despite anatomical differences. In humans, the canalicular stage begins at 16 and ends at 24 weeks of development. By week 20–22, type I and II alveolar cells have differentiated from their progenitors.

Several key transcription factors and signaling pathways are known to regulate the transitions in lung development. Comparatively little is known about the role of miRNAs, which are small non-coding RNA molecules (21–24 nt) that regulate the expression of their target genes post-transcriptionally. Most miRNAs are transcribed as primary microRNAs (pri-miRNAs), which are processed by the Drosha complex into precursor miRNAs (pre-miRNAs) with hairpin structures, and finally cleaved by the RNase III enzyme Dicer. The mature miRNAs are incorporated into nucleoprotein complexes called RNA-Induced Silencing Complexes (RISC), which facilitate binding to the untranslated regions of mRNA transcripts with homologous nucleotide sequences. Target mRNAs are thus rendered unstable and are degraded, or alternatively their translation is blocked without degradation [6,7].

MiRNAs play important roles during early and late lung development. Previous studies have profiled miRNA expression at various developmental stages in human and mouse lung morphogenesis, suggesting a degree of evolutionary conservation of miRNA functions [8,9]. It has been shown that early conditional downregulation of *Dicer* in mouse lung epithelium leads to arrested branching and abnormal growth of the epithelial tubes [10]. Furthermore, mice with deletion of the miRNA17~92 cluster die shortly after birth with pulmonary hypoplasia and cardiac defects [11]; overexpression of this cluster produces a phenotype consisting of hyperproliferation of lung epithelial cells with incomplete differentiation [12]. MiR-17 and let-7 were previously found to be the most abundant miRNAs expressed early in the pseudoglandular and at the canalicular stage, respectively, during murine lung development [13]. The let-7 family of microRNAs controls epithelial proliferation and lung branching morphogenesis. Their expression increases as a hallmark of transition from early epithelial branching to late embryonic development, and reaches a maximum in the adult lungs [13,14].

CDH patients with severe lethal lung hypoplasia show morphological signs of arrested or delayed branching morphogenesis in the pseudoglandular stage (5–17 weeks of human pregnancy, E9.5–16.6 days in mouse embryo) [15]. Additionally, impaired vascular development has been observed which is characterized by an apparent premature differentiation of vascular smooth muscle cells to their contractile phenotype [16,17]. These elements suggest that a morphogenetic defect occurs relatively early during lung organogenesis [18]. We hypothesized a defect of the expansion of the undifferentiated progenitor cells after the pseudoglandular stage before reaching terminal differentiation during the canalicular stage (16–25 weeks of human pregnancy, E16.6–17.4 days in mouse embryo, E15-17 of chick development) [19,20].

The purpose of this study was to characterize further which microRNAs are essential in the pseudoglandular to canalicular transition, when the gas exchanging portion of the lung begins its formation from the bronchioles, and to relate the discovered miRNAs to disorders associated with severe lung hypoplasia. In fact, little is known about the genetic control of CDH lung development in comparison to diaphragm formation [21,22]. MiR-449a was found to be highly expressed at the canalicular stage compared to the pseudoglandular stage. We showed that its inhibition increased epithelial differentiation while its overexpression resulted in marked pulmonary hypoplasia, as seen in severe CDH. Furthermore, we found that miR-449a controlled epithelial proliferation in the developing lung.

## Materials and Methods

### Human miRNA Microarrays

Total RNA was obtained from two 9 week old fetal lung samples using TRIzol RNA Isolation Reagents (Life Technologies, Carlsbad, CA) homogenized by successive passages in 18G and 25G needles, and from two 18–20 week pooled human male fetal lungs (Stratagene, La Jolla, CA) (Cat. 540177, Lot. 0450170). Total cDNA was hybridized to Human miRNA Microarrays (V1 and V2, based on Sanger miRbase releases 9.1 and 10.1) (Agilent technologies, Santa Clara, CA). Data analysis was performed using the Linear Models for Microarray Data (limma) package in Bioconductor (www.bioconductor.org). The data discussed in this publication have been deposited in NCBI's Gene Expression Omnibus [23] and are accessible through GEO Series accession number GSE76921 (https://www.ncbi.nlm.nih.gov/geo/query/acc.cgi?acc=GSE76921). Human studies were approved by the Partners Institutional Review Board (IRB) at the Massachusetts General Hospital (Protocols 2000-P-000372 and 2002-P-000083) and CREC no. 2001.201 from the Joint CUHK-NTEC Cluster Clinical Research Ethics Committee.

### Mouse and chick tissue collection

Lungs were harvested from inbred C57/B6 timed-pregnant mice (Charles River, Wilmington, MA) and from timed, fertilized, white leghorn eggs (Spafas Inc, Voluntown, CT) maintained in a humidified incubator (Kuhl Corp, Flemington, NJ) at 38°C. Mouse and chick embryos were staged according to accepted criteria [24,25]. Animal studies were approved by the Center for Comparative Medicine at the Massachusetts General Hospital Institutional Animal Care and Use Committee (Protocol 2012-N-000025).

### Organ Culture

Freshly dissected E16.5 lung 1–2 mm slices were placed in 24-mm Transwell permeable support plates (Corning Inc., Corning, NY) and incubated for two days in BGJB (Gibco | Life Technologies, Carlsbad, CA) medium containing 0.2 mg/ml L-Ascorbic acid (Sigma-Aldrich, St. Louis, MO), 50 U/ml penicillin and 50 U/ml streptomycin (Sigma-Aldrich, St. Louis, MO) at 37°C in 5% CO_2_ [26]. Anti-miRNA-449a and scrambled (control) Peptide Nucleic Acids (PNA) (Panagene, Daejeon, Korea) were transfected using Effectene Transfection Reagent (Qiagen, Venlo, The Netherlands). Tissue samples were processed after four days.

### *In ovo* viral transduction

A replication-competent avian specific retrovirus (RCAS; A coat) was engineered to express the RCAS(A)-449a construct composed of the miR-449a murine premiR sequence flanked by 200 nucleotides, using established techniques, and grown and harvested in DF1 cells [27,28]. Embryos were injected, *in ovo*, at E2 (st11-15) in the right anterior-lateral region targeting the pre-lung field, following a published fate map, with approximately 1μl of freshly defrosted virus [29]. Injections were carried out under a Nikon SMZ800 dissection microscope, using a Hamilton syringe fitted with pulled glass micropipette needles [28]. Eggs were then sealed and returned to the incubator until harvesting at E10, E13, E15, or E17. More than 15 dozen embryos were injected with *RCAS(A)*-*449a*. Controls consisted of sham injections and *RCAS(A)-GFP*-injected embryos.

### Quantitative RT-PCR

Total RNA was extracted from pooled mouse (N = 6) and chick (N = 3) embryonic lungs using TRIzol RNA Isolation Reagents (Life Technologies). RT-qPCRs were performed using iQ SYBR Green Supermix (Bio-Rad Laboratories, Waltham, MA). In miRNA experiments, cDNA was synthesized from 1ug of total RNA by miScript PCR Starter Kit (Qiagen, Venlo, The Netherlands); real-time data were generated using miR-449a miScript Primer Assays and normalized against snRNA RNU6B (RNU6-2) (Qiagen, Venlo, The Netherlands). In mRNA experiments, cDNA was synthesized from 1ug of total RNA by SuperScript First-Strand Synthesis System (Invitrogen | Life Technologies). Real-time data were generated with primers designed using National Center for Biotechnology Information (NCBI) Primer-BLAST. *Actb* was selected as the most stable normalizer determined by Biogazelle qbase+ (www.biogazelle.com). Human adult RNA was obtained from four pooled female donors, ages 28–66 (Stratagene, La Jolla, CA) (Cat. 540019, Lot. 0960449). Total RNA from freshly prepared sections of formalin-fixed paraffin-embedded human fetal tissue was extracted with the Pinpoint Slide RNA Isolation System II (Zymo Research Corporation, Irvine, CA). Retrotranscription and real-time PCRs were performed as described above. Results were analyzed using the ΔΔC_t_ method, and significance calculated by Student's *t*-test.

### Immunohistochemistry (IHC) and *In Situ* Hybridization (ISH)

Mouse and chick lungs were dissected and fixed with 4% paraformaldehyde (PFA) or 10% formalin in RNase-free PBS, respectively, for 2 hours at 4°C. Fixed tissues were washed in PBS with 0.1% Tween 20 (PBT) and through a graded series of methanol/PBT, or maintained at -20°C in methanol until use. Paraffin sections were heat treated in a microwave oven at medium power in 0.01 M sodium citrate buffer (pH 6) for 20 minutes for antigen retrieval. Before antibody incubation, peroxidase was quenched with H_2_O_2_. Biotinylated secondary antibodies (Vector Laboratories, Burlingame, CA) were used to localize antibody-antigen complexes. Antigen detection was performed with the ABComplex/HRP Detection System (DakoCytomation, Glostrup, Denmark), following the manufacturer's directions, and enhanced with 3,3′-Diaminobenzidine (DAB). The following antibodies were used in this study: mouse anti-PCNA (1:150; NeoMarkers; Fremont, CA;, USA), anti-NKX2.1 (1:200; mouse monoclonal; SantaCruz, Dallas, TX, USA), and anti-SOX9 (1:200; rabbit polyclonal; a gift from Dr. de Santa Barbara, University of Montpellier, France), anti-N-MYC (1:200; rabbit polyclonal; abcam; Cambridge, MA, USA), anti-SOX2 (1:200; rabbit polyclonal; abcam; Cambridge, MA, USA), anti-pSPC (1:400; rabbit polyclonal; abcam; Cambridge, MA, USA) and anti-Ki67 (1:150; rabbit monoclonal; abcam; Cambridge, MA, USA). Predesigned LNA-enhanced microRNA ISH Detection Probes were used according to the manufacturer's directions (Exiqon, Vedbaek, Denmark).

### Luciferase Assay

A vector with a basal promoter and the luc2P gene inserted upstream of the human *MYCN* 3’ UTR (SwitchGear Genomics) and premiR-449a or scrambled premiRs (Ambion | Life Technologies, Carlsbad, CA) were co-transfected (Effectene, Qiagen, Venlo, The Netherlands) in the immortalized Human Embryonic Kidney 293 (HEK) cells (ATCC) in Dulbecco’s Modified Eagle Medium (Gibco | Life Technologies, Carlsbad, CA), 10% fetal bovine serum, and 1% penicillin/streptomycin. Cells were cultured at 37°C and 5% CO_2_ saturation. QuikChange Site-Directed Mutagenesis Kit (Stratagene, La Jolla, CA) was used to delete either one or both predicted miR-449a binding sites within the N-myc 3’ UTR in the previously described luciferase vector. The Dual-Luciferase Reporter Assay System (Promega, Madison, WI) was used to assess luciferase activity, normalized to background renilla activity. The reported measurements are representative of three or more experiments, conducted in six-plicate in 96 well plates. Significance was calculated by Student's *t*-test.

## Results

### miR-449a is expressed during midgestation in human, murine, and avian lungs

**Fig 1:**
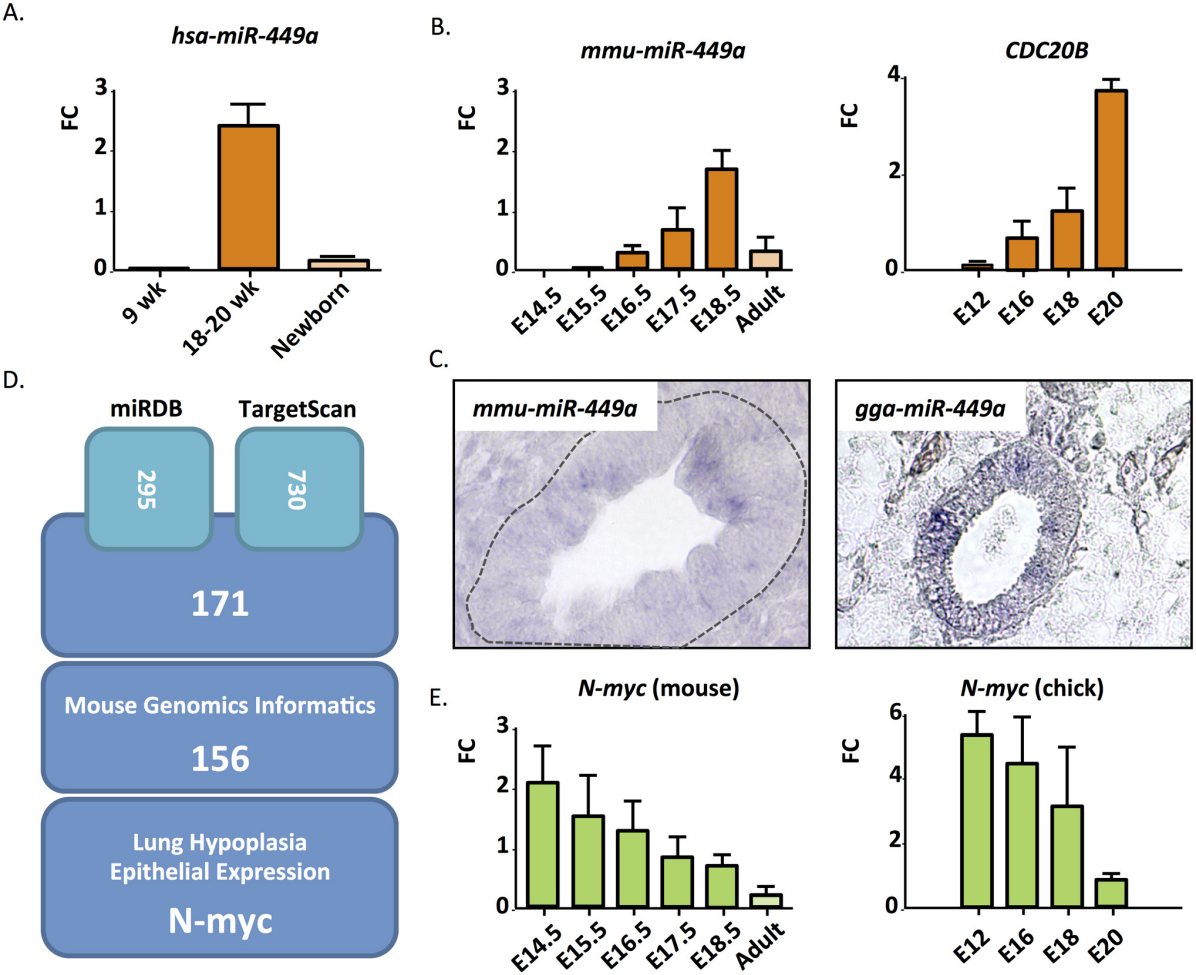
Mir449a expression. **A.** Hsa-miR-449a is highly expressed at 18–20 weeks (canalicular) relative to 9 weeks (pseudoglandular) and newborn human human lungs. **B.** Mmu-miR-449a, and CDC20B as a proxy for chick gga-miR-449a, in mouse and chick lungs. **C.** MiR-449a expression in mouse (E15.5) and chick (E12) distal lung epithelium by LNA ISH. Expression was not detected in the mesenchyme. **D.** N-myc is the only predicted target of miR-449a associated with lung hypoplasia and expressed in the lung epithelium.**E.** N-myc expression during mouse and chick lung development is anticorrelated with that of miR-449a. Standard Error is indicated. *FC*, Fold Change.

Human fetal lung specimens at 9 and 18–20 gestational weeks were used to determine miRNA expression changes during the transition from the pseudoglandular to canalicular phases of lung development. Linear Models for Microarray Data (limma) analysis identified 100 up-regulated microRNAs in the canalicular phase. MiRNA-449a exhibited the greatest increase in expression among differentially expressed genes, confirmed by real-time qPCR (Fig 1A) and replicated in C57BL/6 mouse embryonic lungs at comparable developmental stages (Fig 1B). It is worth noting that human hsa-miR-449a and its murine ortholog mmu-miR-449a-5p share identical mature sequences in miRBase (www.mirbase.org). Interestingly, miRNA-449a reached its peak level of expression at 18 weeks (*H*. *sapiens*), or E18.5 (*M*. *musculus*), corresponding to the final stages of canalicular development. Finally, its expression declined in the saccular stage and was minimal at birth suggesting time specific function in that critical stage of lung development. MiR-449a, and its paralogs miR-449b and miR-449c, are co-regulated with their host gene CDC20B [30]. During chick (*G*. *gallus*) lung development, *CDC20B* expression was highest at E18 by RT-qPCR of whole-lung extracts (Fig 1B), suggesting that the developmental control of miR-449a expression follows the same pattern in mammals and avians. MiR-449a is expressed al low levels in the distal lung epithelium, and not in the mesenchyme, of mouse and chick lung explants (Fig 1C).

### *Mycn* transcripts are regulated by miRNA-449a

Several transcripts were predicted as putative hsa-miR-449a targets by TargetScanHuman Release 6.2 (N = 655; 730 conserved binding sites in the miRNA family) (www.targetscan.org), and by miRDB (N = 295) (mirdb.org). Out of the 171 predictions in common between the two databases, 156 were annotated in the Mouse Genome Informatics web portal (informatics.jax.org). We chose to investigate *N-Myc* [v-myc myelocytomatosis viral related oncogene, neuroblastoma derived (avian)] which is associated, when perturbed, with abnormal branching similar to the lung morphogenesis defect and the pulmonary hypoplasia characteristic of severe and lethal CDH (Fig 1D).

The negative correlation (anticorrelation) between N-Myc and miR-449a expression was confirmed by RT-qPCR in human and mouse lung samples (Fig 1E). N-Myc and *CDC20B* expression levels were similarly correlated in chick lungs (Fig 1E).

### Hsa-miR-449a binds to the *MYCN* 3’-UTR

**Fig 2:**
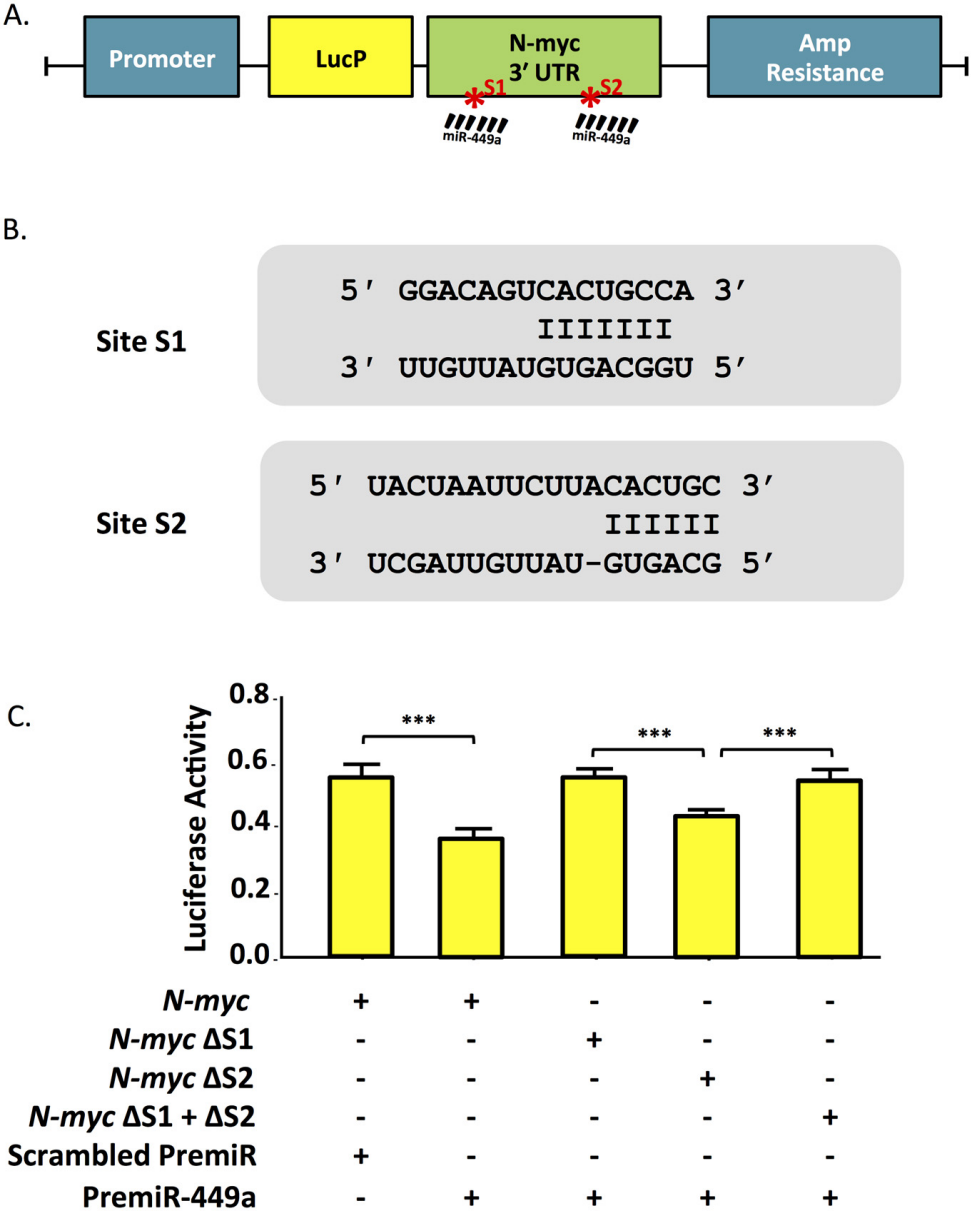
**A.** Diagram of N-myc 3' UTR luciferase reporter vector. MiR-449a predicted binding sites S1 and S2 are indicated. **B.** Site S1 and S2 sequence aligned with miR-449a. **C.** PremiR-449a reduces N-myc 3' UTR luciferase activity relative to scrambled control. S1, but not S2, deletion restores luciferase activity.

Luciferase assays based on a vector with a basal promoter and the luc2P gene upstream of the N-myc 3’UTR were used to confirm regulation by the miR-449a (Fig 2A). The construct contained both predicted binding sites S1 and S2 (Targetscan 5.1) (Fig 2B). The luciferase vector, a renilla vector for transcription control, and either pre-miR-449a or a negative control scrambled pre-miRNA were co-transfected into HEK cells, chosen from a panel of cell lines because they exhibited the lowest endogenous miR-449a expression in order to maximize signal-to-noise.

Pre-miR-449a transfection caused a significant reduction in luciferase activity compared to scrambled pre-miRNA treated cells (p < 0.0001), indicating direct regulation of the N-myc 3’UTR by miR-449a (Fig 2C). The two binding sites were then deleted in the luciferase/N-Myc 3’ UTR vector by site directed mutagenesis, resulting in three new vectors with Site 1 (S1), Site 2 (S2), or S1+S2 targeted deletions. Their co-transfection with pre-miR-449a resulted in abrogation of the miR-449a effect when either S1 or S1+S2 were deleted, whereas deletion of S2 alone had only a modest effect on luciferase activity, indicating that S1 in the N-Myc 3’ UTR is the critical binding site for miR-449a (Fig 2C).

### miR-449a regulates epithelial proliferation and differentiation *ex vivo* and *in ovo*

**Fig 3:**
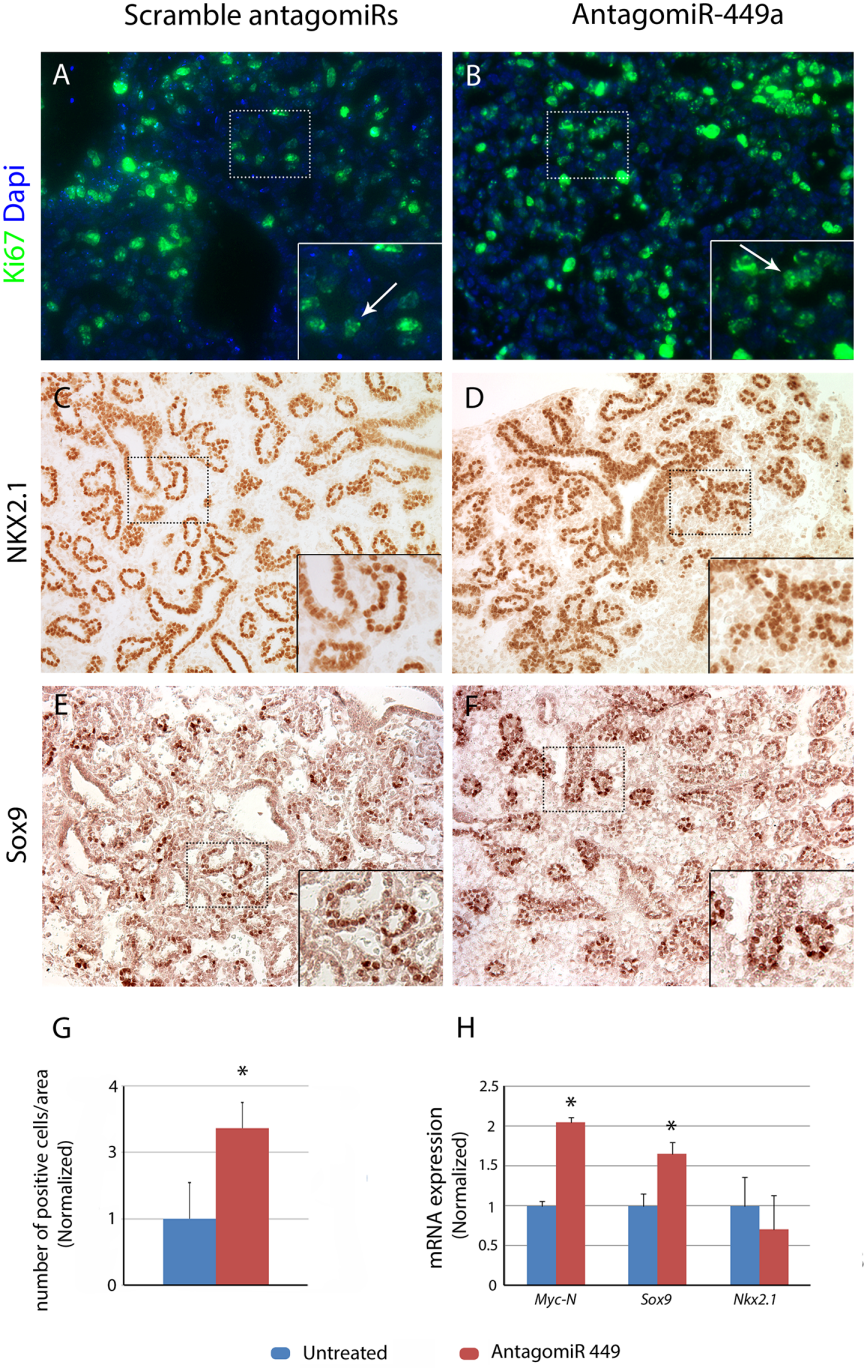
Mouse ex vivo lung culture. Right middle lobe organ culture with scrambled sequence antagomirs (Control; A,C,E) and antagomiR-449a (B,D,F), stained with anti-Ki-67 (A,B), anti-NKX2.1 (C,D), and anti-SOX9 (E,F) antibodies. Inhibition of MiR-449a increases SOX9 expression. Insets: 40X magnification. G. ImageJ counts of SOX9 positive cells in histological sections of antagomiR treated lung organ cultures, relative to untreated controls. H. Real-time qPCR results show increased *Mycn* and *Sox9* expression in antagomiR treated lungs (p<0.05, two-tailed t-test with unequal variances, aggregate results of 3 separate experiments). *Nkx2*.*1* levels were not significantly altered.

By competitive binding of PNA antagomirs, we investigated whether functional knockdown of miR-449a affected lung epithelial progenitors in *ex vivo* organ cultures. Lungs were harvested from E16.5 embryos, corresponding to the end of the pseudoglandular phase, at the onset of miR-449a expression. PNA antagomirs added to the culture media effectively increased N-Myc mRNA levels after 48 hours. Expression of both the proliferative marker Ki-67 (Fig 3A and 3B) and the epithelial progenitor marker SOX9 (Fig 3E, 3F and 3G) were increased in the distal portion of the epithelium. NKX2-1 (Fig 3C and 3D), pSPC (S1 Fig), and Sox2 (S1 Fig) expression was not altered. These findings were confirmed by RT-qPCR (Fig 3H).

Given the observed increased epithelial proliferation in antagomir treated mouse lung explants, we hypothesized that miR-449a overexpression would disrupt lung growth, resulting in lung hypoplasia. Therefore, we developed and injected *in ovo* an *RCAS(A)-449a* virus, expressing the murine miR-449a, or the negative control *RCAS(A)-GFP* along with sham injections at the beginning of lung development of chick embryos (E2). Successful infection of the epithelium at E3 was confirmed and monitored by RT-qPCR of the virion component, src, and by AMV-3C2 (anti-gag) antibody staining in the lung buds and throughout development (S1 Fig). Samples with predominant infection of the mesenchyme were not analyzed.

**Fig 4:**
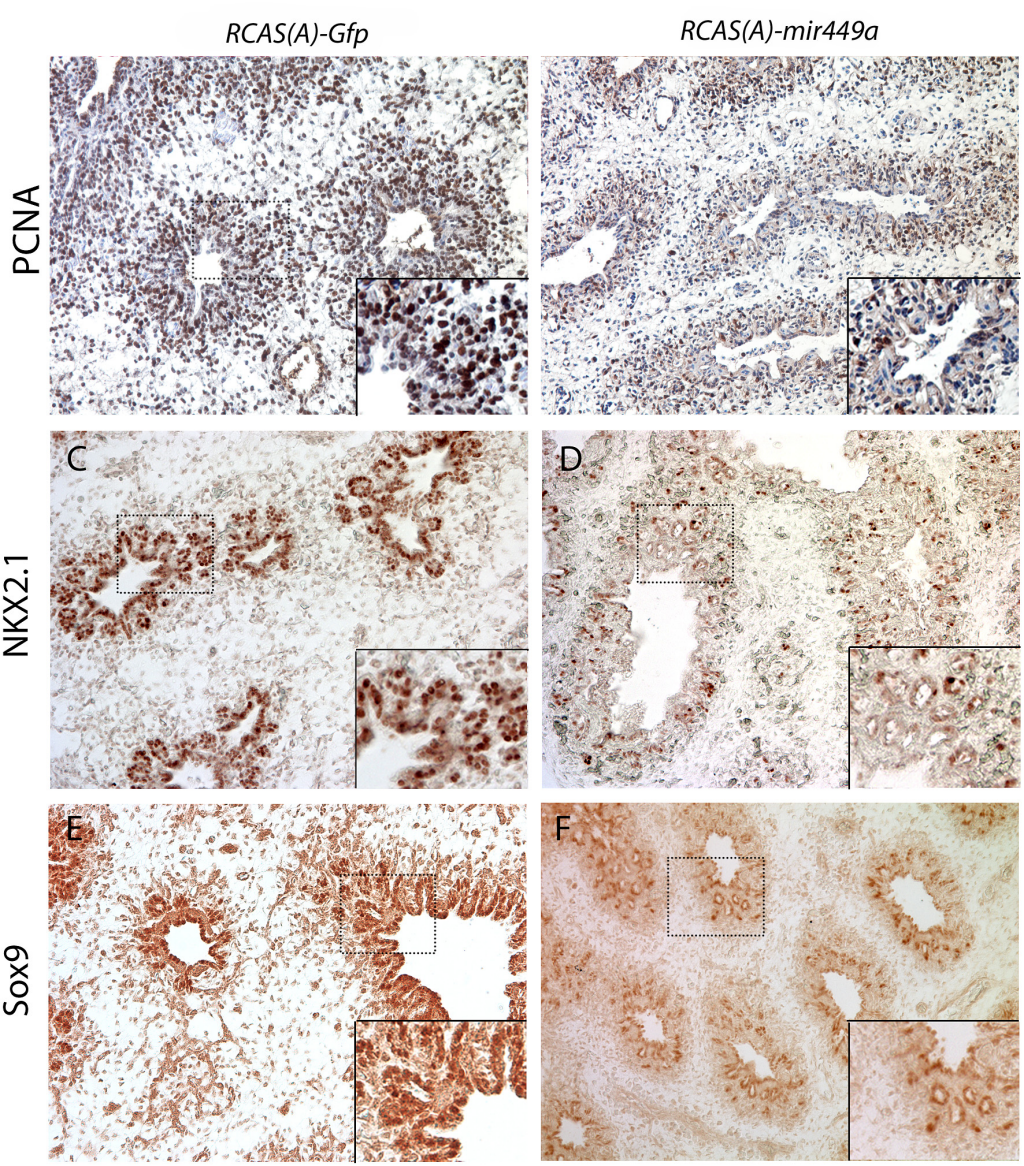
In ovo injections. E15 chick lungs after *RCAS(A)-Gfp* (A,C,E) or *RCAS(A)-mir449a* (B,D,F) *in ovo* infection, stained with anti-PCNA (A,B), anti-NKX2.1 (C,D), and anti-SOX9 (E,F) antibodies. MiR-449a overexpression reduces PCNA, NKX2.1, and SOX9 expression. Insets: 40X magnification.

Early embryonic lethality was observed; however, the lungs of surviving embryos showed marked lung hypoplasia. Moreover, histological examination of the chick E13 lungs showed that *RCAS(A)-449a* infection led to a decreased number of airways. Next, we studied the effect of miR-449 overexpression on lung epithelial proliferation. In less severely affected E15 *RCAS(A)-449a* embryos the proliferative marker, PCNA, was markedly decreased both in the mesenchyme and in the epithelial cells compared to the controls, in which PCNA was abundantly expressed in and around the distal epithelium (Fig 4A and 4B). The epithelial progenitor marker, SOX9, was markedly reduced in the distal airways (Fig 4E and 4F). In general, the entire epithelium was affected as indicated by globally reduced Nkx2.1 expression, indicating that terminally differentiated cells were also compromised (Fig 4C and 4D).

### Hsa-miR-449a expression in hypoplastic CDH lungs

**Fig 5:**
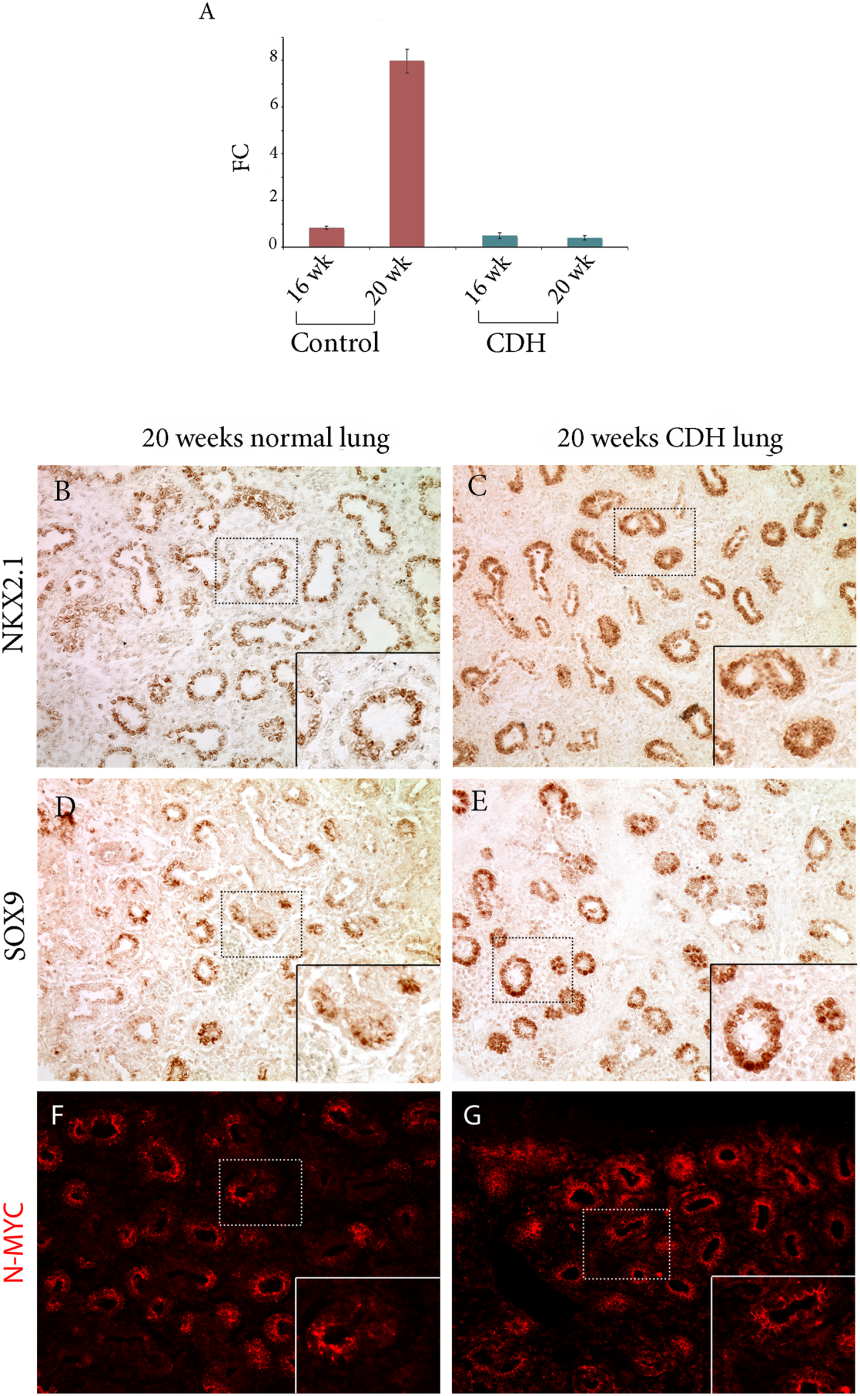
CDH lungs. A. hsa-miR-449a expression increases from 16 to 20 wk in human lungs (left) (n = 2 and n = 1, respectively), but not in patients with CDH (right) (n = 1 and n = 1, respectively). B-E. NKX2.1 (B,C) and SOX9 (D,E) expression revealed by immunohistochemistry. F-G. N-MYC expression is increased and more widely distributed in distal epithelium of CDH lungs.

As pulmonary hypoplasia is associated with diaphragmatic defects in humans and in animal models, we assessed miR-449a expression levels in lung paraffin embedded specimens from available CDH fetuses by RT-qPCR. MiR-449a expression differences were not detectable at the onset of the canalicular phase between a 17 week CDH specimen (N = 1) and 16 week reference samples (N = 2) (Fig 5A). In the mid-late canalicular phase, at 20 weeks gestation, miR-449a appeared to be reduced in a CDH fetus (N = 1) relative to a reference sample of the same gestational age (N = 1) (Fig 5A). NKX2.1 expression was not altered in these samples (Fig 5B and 5C), while SOX9 expression was increased (Fig 5D and 5E). In addition, N-MYC expression in CDH lungs was increased and more widely expressed thorough the distal tips of growing airways in diseased lungs (Fig 5F and 5G) This phenotype is consistent with reduced miR-449a levels, possibly correlated with lung immaturity. These data, necessarily based on the limited samples available for research purposes, suggest a role for hsa-miR-449a in the pulmonary phenotype of CDH patients prenatally, which should be further explored.

## Discussion

Microarray expression profiles from human lung tissue in the pseudoglandular and canalicular stages of lung development revealed several differentially expressed miRNAs. The most highly upregulated miRNA was miR-449a, confirming published microarray data in mice [31]. MiR-449a is a critical regulator of genes involved in cellular proliferation, differentiation, and apoptosis [30,31]. In fact, miR-449a expression is normally controlled by the transcription factor E2F1, a potent stimulator of cell cycle progression [31,32].

In the present study, quantitative PCR revealed a time specific increase in expression of miR-449a at E15.5-E18.5, which corresponds to the end of branching morphogenesis in the late pseudoglandular phase, and throughout the canalicular phase; its expression decreased dramatically at birth. This pattern suggests a specific role for miR-449a in the mid stages of lung development. In the lung, upregulation of the miRNA-449a had been correlated with the differentiation of ciliated cells in proximal pulmonary epithelia through the Delta/Notch pathway [30,33]. Mucociliary differentiation largely occurs late in lung development; our data, however, support the hypothesis that miR-449a may additionally influence distal epithelial progenitor proliferation.

A prioritized list of likely miRNA-449a targets was obtained through a compilation of the online tools TargetScanHuman Release 6.2 and miRDB. The MGI database was queried to identify which of the target genes were known to cause pulmonary hypoplasia in mouse models. N-myc, a transcription factor belonging to the myc basic Helix-Loop-Helix DNA binding domain family, was the only predicted miR-449a target in the MGI database to be associated to both abnormal branching morphogenesis and pulmonary hypoplasia in mouse models, and also expressed in lung epithelial cells like miR-449a. Its paralog c-myc is a proto-oncogene with a role in several human cancers including Burkitt’s lymphoma [34]. Similarly, N-myc plays a role in tumorigenesis; for example, neuroblastoma patients with N-myc overexpression are at increased risk of metastasis and mortality, and small cell carcinomas of the lung are also known to overexpress N-myc in certain subtypes [35–37].

Homozygous deletion of N-myc results in embryonic lethality at E11.5. Null embryos demonstrated a normal appearance and formation of early primordial organs, but failure of subsequent proliferation resulted in severe hypoplasia of the lungs, genitourinary system, gut, and parts of the nervous system [38]. Heterozygous mice were also less viable than wild-type and exhibited impaired fitness [38]. Conversely, N-myc overexpression in utero results in a lung phenotype consisting of hyperproliferation and decreased differentiation, resulting in distal epithelial tubules surrounded by abundant mesenchyme [39]. Transgenic pups with conditional overexpression of the miRNA 17~92 cluster in the fetal lung similarly die soon after birth or have severe respiratory distress, and manifest increased epithelial cell proliferation and increased expression of Sox9 and N-myc in the lungs [12]. Further, overproliferation of Sox9-positive cells was also described in nitrofen-treated lung explants, a validated model of CDH-associated lung hypoplasia [40].

Thus, N-myc plays an important role in embryonic lung development and the data presented herein point to a role for miR-449a as a regulator of N-myc, as confirmed by luciferase assays. Site directed mutagenesis experiments with a series of 7 nucleotide deletions of the miR-449a:N-myc binding site, confirmed a direct interaction. Specific deletion of the 5’ S1 predicted binding region alone was sufficient to result in significant loss of the miR-449a effect, indicating that S1 is the functionally active binding site.

By the time miR-449a can be first detected by our methodology, proximal cells arising from a pool of epithelial progenitors, have already begun to abandon their undifferentiated state [41]. Specifically, the reduction in their proliferation rate is an absolute requirement for correct differentiation, indicating that the balance between proliferation and differentiation is tightly controlled [42]. The miR-449a target N-myc belongs to the group of genes that maintain the proliferation of undifferentiated progenitors [39]. Similarly, the pool of Sox9 expressing progenitors is expanded when miR-449a is antagonized, or reduced when it is overexpressed. Therefore, we speculate that the N-myc regulation operated by miR-449a may be one of several cellular mechanisms used by epithelial progenitors to escape their undifferentiated proliferative state and thus coordinate the critical process of epithelial differentiation. Distal populations of epithelial progenitors start to express miR-449a as they leave the tip of the airways. MiR-449a expressing cells then engage two separate pathways, the first to reduce their proliferative rate, the second to initiate proximal epithelial mucociliary differentiation [30].

In conclusion, although several candidates have emerged from this unbiased microarray screen between the pseudoglandular and canalicular phases of lung development, miR-449a stood out as the most significantly upregulated, with N-myc as a likely target in the epithelium at this time in development according to prediction algorithms, the MGI database of mouse phenotypes, and luciferase assays. Furthermore, miR-449a murine *ex vivo* functional knockdown and avian *in ovo* overexpression documented morphological changes consistent with impaired lung differentiation and proliferation. Precise gene regulation during lung development is critical for proper organogenesis; consequently, its disruption may be a feature of human disorders characterized by pulmonary hypoplasia. Dynamic regulation of N-myc by miR-449a is a promising interaction to investigate further as a likely mediator of epithelial cell differentiation. Additionally, we detected abnormal miR-449a expression in the necessarily limited human CDH samples available for research purposes. N-MYC expression was increased in the available CDH lung sample. Interestingly, at least one patient with a *MYCN* mutation has been reported to have CDH [43]. Although we do not presently know whether decreased miR-449a expression is sufficient to cause the full human phenotype or how it affects the diaphragm, the present study suggests that miR-449a dysregulation plays a role in the pathophysiology of CDH-associated lung hypoplasia.

## Supporting Information

S1 Fig A-D. Mouse lung explants and *ex vivo* organ culture. Sox2 and pSPC positive cells were identified by IHC in scrambled sequence antagomirs as controls (A, C), or treated with antagomir-449a (B, D). E-F. Expression of the 3C2 viral marker was measured in *RCAS-mir449* infected chick samples (*, lung airways or parabronchi).
(TIF)
