# Supplementary figures and images for: MiR-449a Affects Epithelial Proliferation during the Pseudoglandular and Canalicular Phases of Avian and Mammal Lung Development
Source: PLoS One. 2016 Feb 18;11(2):e0149425. doi: 10.1371/journal.pone.0149425 (PMC4758652; doi:10.1371/journal.pone.0149425)

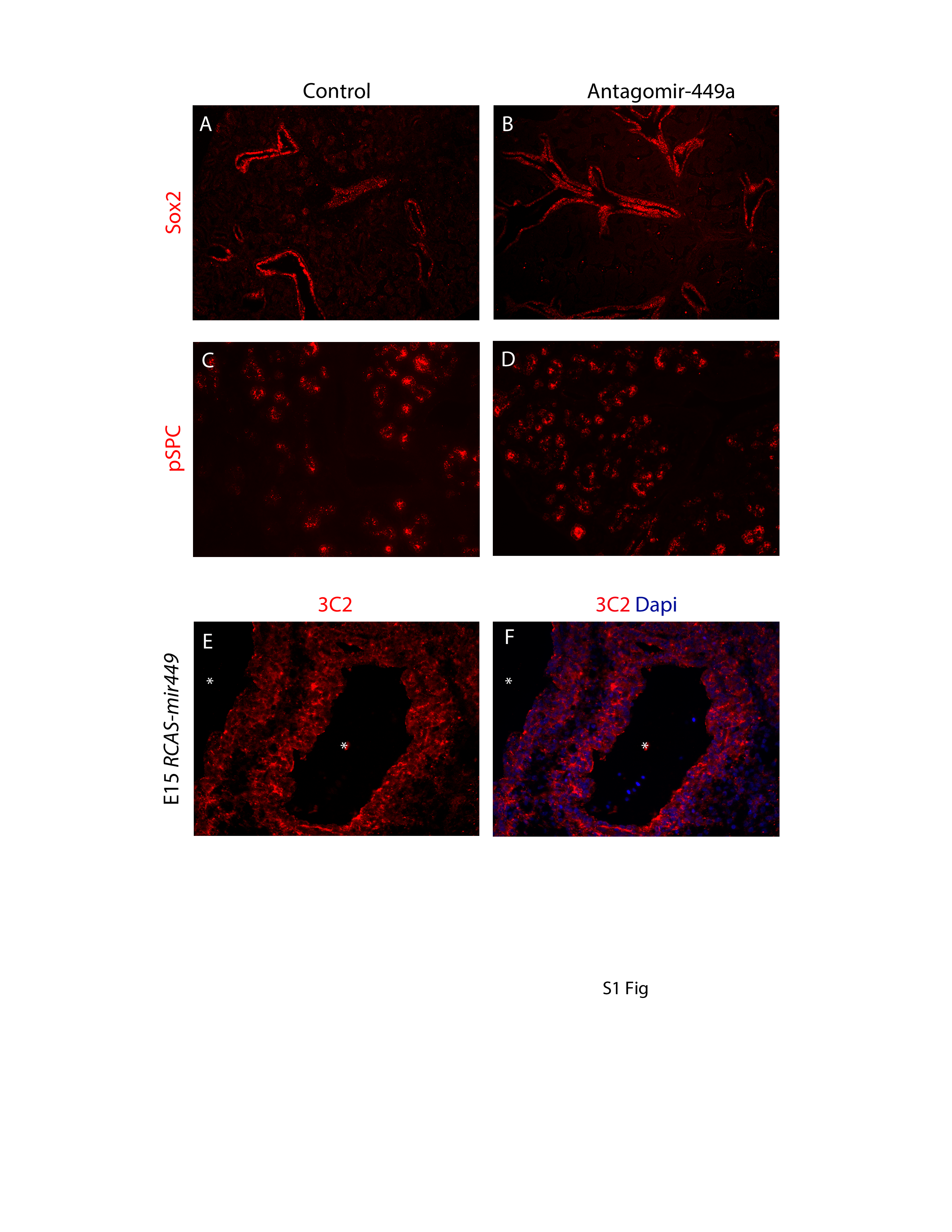

Supplement: S1 Fig — A-D. Mouse lung explants and ex vivo organ culture. Sox2 and pSPC positive cells were identified by IHC in scrambled sequence antagomirs as controls (A, C), or treated with antagomir-449a (B, D). E-F. Expression of the 3C2 viral marker was measured in RCAS-mir449 infected chick samples (*, lung airways or parabronchi). (TIF) [file pone.0149425.s001.tif]
